# A Smartphone App Self-Management Program for Chronic Obstructive Pulmonary Disease: Randomized Controlled Trial of Clinical Outcomes

**DOI:** 10.2196/56318

**Published:** 2025-04-23

**Authors:** Lisa Glynn, Eddie Moloney, Stephen Lane, Emma McNally, Carol Buckley, Margaret McCann, Catherine McCabe

**Affiliations:** 1 School of Nursing and Midwifery University of Galway Galway Ireland; 2 Tallaght University Hospital Dublin Ireland; 3 Trinity College Dublin Ireland

**Keywords:** COPD, self-management, smartphone application, clinical health outcomes, chronic obstructive pulmonary disease, clinical health, patient, patients, randomised controlled trial, RCT, smartphone, app, apps, application, applications, mhealth, mobile health, effectiveness, intervention, interventions, hospital setting, quality of life, self-efficacy, hospital, hospitals, mobile phone

## Abstract

**Background:**

Chronic obstructive pulmonary disease (COPD) negatively impacts clinical health outcomes, resulting in frequent exacerbations, increased hospitalizations, reduced physical activity, deteriorated quality of life, and diminished self-efficacy. Previous studies demonstrated that a self-management program tailored for adults with COPD improves self-management decisions, resulting in a positive effect on clinical health outcomes. Limitations of these studies include issues regarding heterogeneity among interventions used, patient population characteristics, outcome measures, and longitudinal studies. Limited studies focused on the use of a comprehensive self-management program using a smartphone app for adults with COPD over 12 months.

**Objective:**

This study aimed to explore the effectiveness of a smartphone app self-management program and monthly phone calls compared with standard respiratory outpatient care on clinical health outcomes in adults with COPD.

**Methods:**

This was a 3-arm parallel pilot randomized controlled trial (RCT) that included 92 participants. Participants were randomized into intervention arm 1, which included a self-management smartphone app and monthly phone calls (n=31); intervention arm 2, which included a self-management smartphone app (n=31); and arm 3, which was standard respiratory outpatient care (n=30). All arms received standard respiratory outpatient care. The primary outcome was a binary indicator equal to 1 if participants reported attendance to a general practitioner (GP) and or a hospital setting as a result of an exacerbation and 0 otherwise. This indicator was recorded at 6 months and 12 months from the baseline. Secondary outcomes included engagement, breathlessness, physical activity, health-related quality of life, and self-efficacy.

**Results:**

There was a statistically significant difference (*P*=.03), indicating fewer exacerbations in the intervention arm 2 compared with the control arm at 6 months in the hospital setting. The intervention arms had a statistically significant difference indicating a lower risk of developing an exacerbation at 6 months in both the GP (*P*=.01) and hospital setting (*P*=.006) compared to the control arm. Furthermore, intervention arm 1 demonstrated a statistically significant difference in exercise capacity at 6 and 12 months (*P*=.02 and *P*=.03). The intervention arm 2 illustrated a statistically significant difference in step count (*P*=.009) compared to the control arm. The majority of participants (60%, 33/55) used the app over the 12-month period.

**Conclusions:**

This study demonstrated that a smartphone app self-management program had a positive effect on clinical health outcomes for participants with COPD in comparison to standard respiratory outpatient care. This study illustrated benefits such as reduced exacerbations resulting in fewer hospitalizations, improved exercise capacity, and physical activity among the intervention arms. This was a single-center study, which was limited in power to demonstrate significant effects on all measured outcomes but paves the way for a larger, fully powered multicenter trial exploring the effect of a smartphone app self-management program on clinical health outcomes in adults with COPD.

**Trial Registration:**

ClinicalTrials.gov NCT05061810; https://clinicaltrials.gov/study/NCT05061810

## Introduction

### Background

Chronic obstructive pulmonary disease (COPD) is a progressive lung disease resulting in persistent respiratory symptoms, such as a chronic productive cough, breathlessness, wheezing, and airflow limitation primarily caused by cigarette smoking and other noxious gases. It is characterized by a chronic decline in lung function with irreversible airflow obstruction and systemic manifestations resulting in frequency and severity of exacerbations. COPD exacerbations are defined as acute events described by a worsening of respiratory symptoms that are beyond normal day-to-day variations [1,2]. COPD exacerbations are the most frequent presentation in the hospital setting, both nationally and internationally. This accounts for a substantial economic burden [2,3]; for instance, within the European Union, COPD accounts for 56% of the overall respiratory costs annually [4]. COPD can negatively impact clinical health outcomes, resulting in frequent exacerbations, increased hospitalizations, reduced physical activity, deteriorating quality of life, and a diminished sense of self-efficacy [1,2,5-9].

Self-management programs have been shown to improve individual’s knowledge, confidence, and skills to self-manage their chronic illness [1,10]. This results in improved clinical health outcomes in terms of enhanced quality of life, improved physical activity, and fewer exacerbations, resulting in reduced hospitalizations, morbidities, and premature death among this cohort [1,7-9]. Successful self-management interventions among adults with COPD result in cost-effectiveness for the health care service [10-14]. Previous studies using self-management programs through a smartphone app have shown a positive effect on clinical health outcomes among this cohort [15-23]. However, these studies [15-28] have issues with heterogeneity among interventions used, consistency of their application, patient population specifics, duration of studies, and outcome measures. In addition, studies have suggested that support from a third party, such as a health care professional may improve engagement levels [16,21,23,24,28]. However, there is no conclusive evidence to support that a third party, such as a health care professional, has a statistically significant impact on engagement levels among this cohort. Therefore, there is a strong need for more research surrounding smartphone apps delivering a comprehensive self-management program for adults with COPD to better understand its role in health care. Furthermore, there is a need to explore whether the use of a third-party involvement, such as a health care professional, to support trial participants in using the app, has any impact on engagement levels.

The use of smartphone apps, remote monitoring, and telemedicine are more frequently used in the delivery of health care both nationally and internationally than before the COVID-19 pandemic [29,30]. It has also been reported that the COVID-19 pandemic has positively influenced the older population’s views on technology, resulting in increased use of smartphones following the pandemic [30,31]. Readily available access to educational resources can be a challenge for participants with COPD due to cost, environmental barriers, timing not being suitable, or a lack of transport to travel to these educational sessions [32]. Smartphones have many benefits, such as providing convenient communication with the patient and health care professional; they offer portability, Bluetooth, and internet connection, allowing for the use of various smartphone apps to work anywhere at any time. In addition, smartphone apps support behavior changes by providing education, interactive feedback, motivational messages, and access to internet-based resources available at any time. Also, they are generally available at a lower cost in comparison to other digital technologies, such as computers or tablets [13,33,34]. However, technology is still evolving [35], and the best practices in relation to smartphone apps supporting a comprehensive self-management program are not yet well established.

We conducted a single-center, 3-arm parallel pilot randomized controlled trial (RCT) to explore the effect of a smartphone app self-management program on clinical health outcomes in adults with COPD on a longitudinal basis of 12 months. The main components of this self-management program were monthly education, symptom tracking, communication with a health care professional, goal setting, and weekly motivational messages from the app. Symptom tracking included monitoring physical activity (step count), breathlessness score (modified Medical Research Council dyspnea scale [mMRC]), and recording of clinical parameters (lung function [forced expiratory volume at 1 second, FEV1] and oxygen saturation [SpO_2_]) using devices such as a spirometer and pulse oximeter. The measurements obtained from the pulse oximeter and spirometer were downloaded through Bluetooth to the self-management app on the participants’ smartphones. This paper provides data about exacerbation rates, engagement, physical activity, breathlessness, health-related quality of life (HRQoL), and self-efficacy.

### Objective

The objective of this study was to explore the effectiveness of a smartphone app self-management program and monthly phone calls compared with standard respiratory outpatient care on clinical health outcomes in adults with COPD.

## Methods

### Overview

As the concept of this trial was the first of its kind in Ireland, it was important to assess its feasibility; therefore, a single-center pilot trial was conducted. Pilot trials are typically not powered studies as they explore a new concept or intervention or, indeed, a trial design where more data is required before progressing to a larger study [36]. A statistical package [37] was used to calculate the number of participants required for a larger national study that resulted in 1888 participants. A CONSORT (Consolidated Standards of Reporting Trials) checklist was completed to guide the design, analysis, and reporting of trial findings (see Multimedia Appendix 1). This trial compared patient outcomes across three arms of the intervention (all in addition to standard respiratory outpatient care): (1) a smartphone app self-management program and monthly phone calls, (2) the smartphone app self-management program alone, and (3) no additional outpatient care. In addition, this trial provides information on the effect size, refusal rates, and attrition rates that will aid in recalculation of the sample size required for the larger multicenter trial.

### Recruitment

Participants who attended the respiratory outpatient department (OPD) and met the inclusion criteria were invited to participate in this study by telephone.

Participants were eligible if they (1) were aged 18 years or older, (2) had a confirmed COPD diagnosis defined as the presence of post-bronchodilator FEV1/FVC<0.70 [1], (3) had COPD whose severity was defined by the Global Initiative for Chronic Obstructive Lung Disease guidelines (2023) [1] were included, (4) were able to give informed consent, (5) had a smartphone and were able to use it, and (6) had good dexterity to use devices such as a handheld spirometer and pulse oximeter.

Eligible participants were provided with a hard copy of the study information pack, which included a cover letter, an information leaflet outlining the study, an informed consent form, and a returning stamped envelope. Participation in this study was completely voluntary. No financial incentives were offered to participate in this study.

Once written consent was received, a trial register form was completed, and the participant was assigned an ID number. Participants were randomized into each arm using a random allocation computer software package called Random Allocation Software 2.0 [38]. Given the nature of the trial, it was not feasible to blind participants and the researchers involved, given the type of intervention, a smartphone app. However, the research team was blinded to the allocation of participants to each arm by using allocation concealment. Allocation concealment was achieved using an independent health care worker who retained the random allocation sequence. This guaranteed adequate allocation concealment by preventing those recruiting and entering participants into the study from knowing the next assignment. The researcher gave the independent health care worker the participant ID, which was then recorded to the next randomization sequence number. Permuted block randomization, block sizes of three (allocation ratio of 1:1:1), was used in this study to ensure equilibrium. Participants were randomized into intervention arm 1, which included the smartphone app self-management program and monthly phone calls (n=31), intervention arm 2 included the smartphone app self-management program (n=31), and arm 3 was standard respiratory outpatient care (n=30). Furthermore, outcome assessors were blinded using an independent advanced nurse practitioner who reviewed and confirmed whether the outcome assessment met the primary case definition.

### Procedures

**Figure 1:**
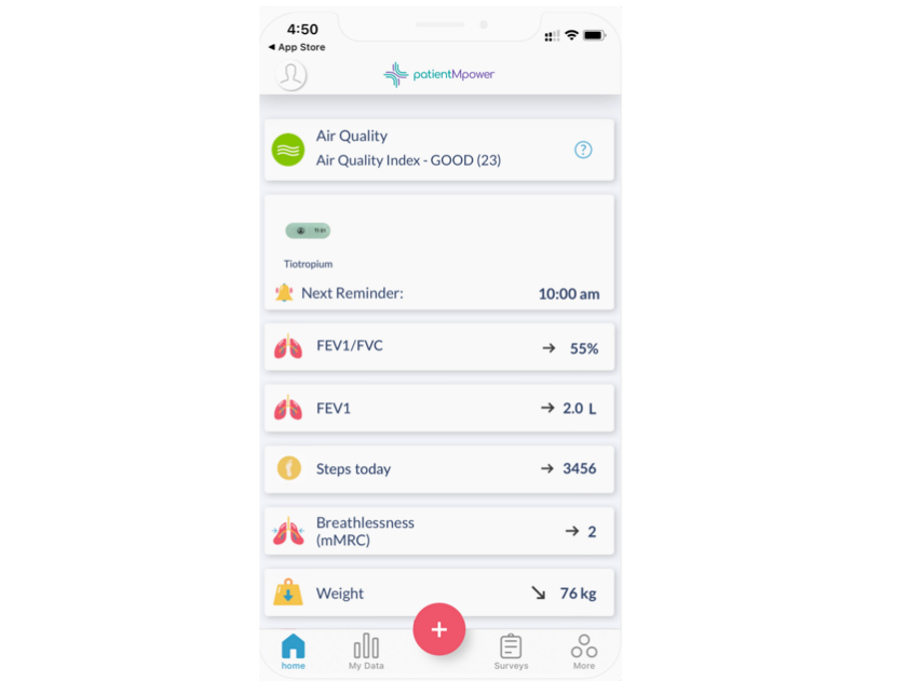
Smartphone self-management app. FEV1: forced expiratory volume at 1 second; FVC: forced vital capacity; mMRC: modified Medical Research Council Dyspnea Scale.

**Figure 2:**
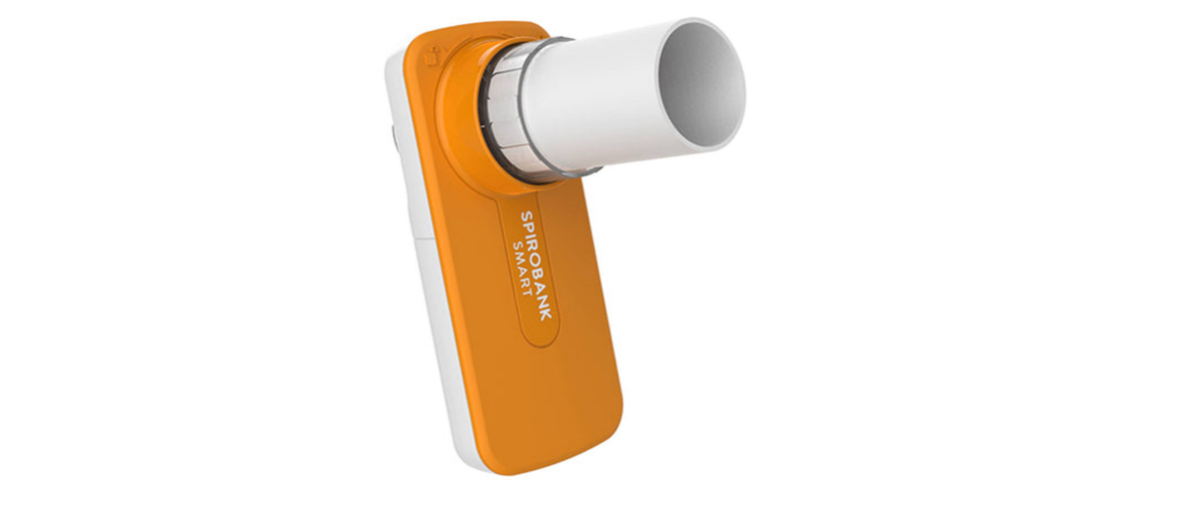
Spirobank smart spirometer.

**Figure 3:**
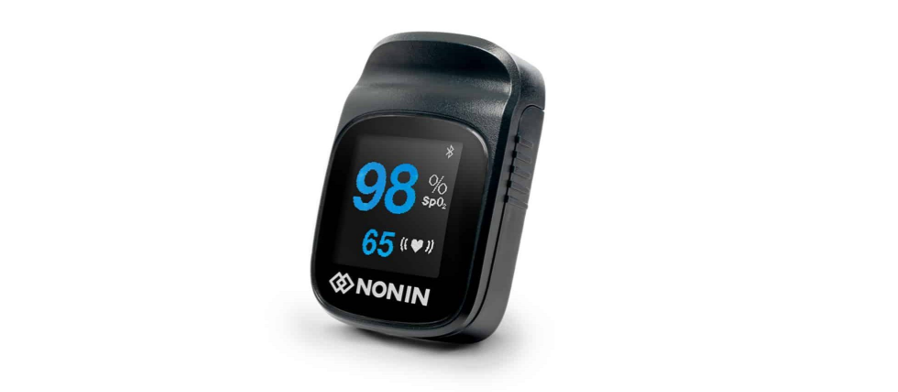
Nonin pulse oximeter.

The intervention in this trial was a comprehensive self-management program through a smartphone app (Figure 1). The main components of this self-management program were monthly education, symptom tracking, communication with a health care professional, goal setting, and weekly motivational messages from the app. Symptom tracking included monitoring physical activity (step count), breathlessness score (mMRC), recording of clinical parameters (lung function [FEV1]), and oxygen saturation (SpO_2_) using devices such as a Spirobank smart spirometer (Figure 2), and a Nonin pulse oximeter (Figure 3) that downloaded via Bluetooth to the self-management program app on the participants' smartphone. In addition, 2 educational videos were uploaded to the self-management app each month for 12 months. Educational videos included features relating to self-management, maintaining a healthy lifestyle, and national support available to participants with COPD.

Participants in the intervention arms were sent an email inviting them to download and activate the self-management program app on their smartphones. This email provided participants with a unique username and password. Education in using the app and devices was provided by the research team and the company patientMpower (pMp). Participants in the intervention arms were provided with a hardback intervention study pack that reiterated the information on how to use the smartphone self-management app and the devices (see Multimedia Appendix 2). Technical support was provided 5 days a week by pMp, and their contact details were displayed in the app and were provided in the intervention study pack. Participants in the intervention arms were advised to use the app by viewing the educational content, input their mMRC score, and use the devices weekly or more times if they wished for 12 months. The smartphone app self-management program sent a motivational message to the participants twice a week, prompting them to use the app. Participants were informed at the recruitment stage that data were collected retrospectively and was not monitored daily. Data were reviewed during the scheduled visits at 6 and 12 months. Participants were informed that the smartphone self-management app was not a replacement for their standard medical care, and in the event of a deterioration in their health status, they should contact their general practitioner (GP) or hospital for medical attention. On completion of the trial, app usage was analyzed to determine participants’ engagement with the app over the 12 months.

Participants allocated to arm 1 received the self-management app along with monthly phone calls from a health care professional and standard respiratory outpatient care. The aim of the monthly phone calls was to provide support to participants using the app. Participants assigned to arm 2 received the self-management app and standard respiratory outpatient care. Participants in arm 3 received standard respiratory outpatient care.

Participants in all the arms of this trial received standard respiratory outpatient care, which consisted of routine visits by telephone at 6 and 12 months from the respiratory OPD at the research site. During these visits, participants informed the research team of any GP and or hospital visits as a result of an exacerbation of COPD. In addition, they informed the team of their physical activity (exercise behavior, capacity, and step count), breathlessness score (mMRC), nutritional intake, pulmonary rehabilitation, medication adherence, smoking status, vaccinations, and if they had contracted the COVID-19 virus. Finally, they completed questionnaires on the burden of symptoms, quality of life, self-efficacy, and user engagement scale (arm 1 and arm 2 only). Participants were also given self-management education and advice during these visits.

It was anticipated that using the self-management app that provided monthly education, motivation, support from a health care professional, and objective data would enhance participants’ knowledge and self-care skills to better manage their chronic illness. This may improve clinical health outcomes, reduce hospitalizations and hospital-associated complications, thereby reducing morbidity, mortality, and overall health care costs among this cohort. The clinical outcomes evaluated in this trial were exacerbation rates, levels of engagement, physical activity, breathlessness, quality of life, and self-efficacy.

### Primary Outcomes

The primary outcome was a binary indicator equal to 1 if participants reported attendance to a GP and or a hospital setting as a result of an exacerbation and 0 otherwise. This indicator was recorded at 6 months and 12 months from the baseline.

### Secondary Outcomes

The first secondary outcome was engagement, which measured viewing the activation rates of the app and devices over 12 months and through the use of a user engagement scale. Also, participants were contacted by phone at 3, 6, and 12 months to complete the refined user engagement scale [39].

Second, physical activity was measured at baseline, 6, and 12 months as the participant reported exercise capacity (participant reported) and step count.

Third, breathlessness was measured using the mMRC scale [40] at baseline, 6, and 12 months. Higher scores represent the severity of breathlessness.

Fourth, the chronic obstructive pulmonary disease assessment test (CAT) [41] score was measured at baseline, 6, and 12 months. Higher scores signified the greater burden of COPD had on the participants’ overall health and well-being.

Fifth, the HRQoL was measured using the clinical COPD questionnaire [42] at baseline, 6, and 12 months. Higher scores illustrated poorer HRQoL.

Sixth, self-efficacy was measured using the Self-Efficacy for Managing Chronic Disease 6-Item Scale at baseline, 6, and 12 months [43]. Higher scores resulted in greater self-efficacy.

### Statistical Analysis

The change in an outcome was considered statistically significant when *P*<.05. All data were analyzed using an intention-to-treat analysis (ITT), resulting in all participants in their allocated arm being followed up to trial completion (12 months). To correlate with the principle of ITT analysis, all participants in this trial were kept in their assigned arms, and sensitivity analysis was conducted. Sensitivity analysis was performed by using the negative outcome for participant attrition (loss of participants to follow up) in the intervention arms (exacerbation of COPD) and participant attrition in the control arm to have the best outcome (no exacerbation of COPD), and this was repeated vice versa. Sensitivity analysis results were compared with the original analysis. This assessed the probable impact of participant attrition, resulting in an unbiased estimate of the true effect of the intervention.

Summary statistics were calculated, with the frequency and the percentages being reported for categorical variables. Continuous variables are presented as mean (SD). Finally, for ordinal variables, median and IQR were reported since they were not normally distributed as determined by the Shapiro-Wilk test. To test for significance between baseline and intervention effectiveness using categorical data, the chi-square, and Fisher exact test were used. Fisher exact test is often used for smaller sample sizes. Independent *t* tests were used to compare the mean difference of continuous outcomes between the intervention (arms 1 and 2) versus the control arm (arm 3). Furthermore, the relative risk and absolute risk reduction, along with the associated 95% CI, were used on all outcomes of this study. The ANOVA test was used for continuous variables (dependent variable) to investigate for a significant difference. In relation to the ordinal variables, the Mann-Whitney *U* test was completed to compare differences between arm 1 and arm 2. The Kruskal Wallis test was completed for ordinal variables to determine a statistically significant difference between the three arms, arm 1, arm 2, and arm 3. The logistic model was used to explore the disparities between the three arms in relation to the primary outcome over time. This provided estimates of the probability of developing a COPD exacerbation over time in each arm, resulting in a GP or hospital visit. The possible influencing factors in developing an exacerbation were included, such as age, gender, time, severity of disease, comorbidities, and smoking history. This data was presented as both adjusted and unadjusted odds ratios (OR) and their corresponding 95% CIs for each variable. In addition, the generalized linear mixed model (GLMM) was used as participants were measured at two different time points, 6 and 12 months. This model is an extension of the logistic regression model, which identifies the correlation exhibited by longitudinal data. The GLMM was used because there were categorical outcomes measured over time resulting in correlated data. The logistic regression model ignores the fact that this data is correlated, thereby ignoring this correlation.

### Ethical Considerations

This study was approved by the St James' Hospital and Tallaght University Hospital Joint Research Ethics Committee, Dublin, Ireland on July 02, 2021. This trial adhered to the rights of the persons, and each participant had the right to make an informed, voluntary decision to participate in this trial. Eligible participants were provided with verbal and written study information packs. Participants had the right to withdraw from this trial at any stage. To adhere to privacy and confidentiality, each participant was assigned a trial identification number and this number was used on all trial documentation. The pseudoanonymized data were stored in a password-protected shared folder at the study center, which was only accessed by the research team. In addition, monetary rewards were not offered for participation in the trial. The ClinicalTrial.gov ID assigned to this study was NCT05061810.

## Results

**Figure 4:**
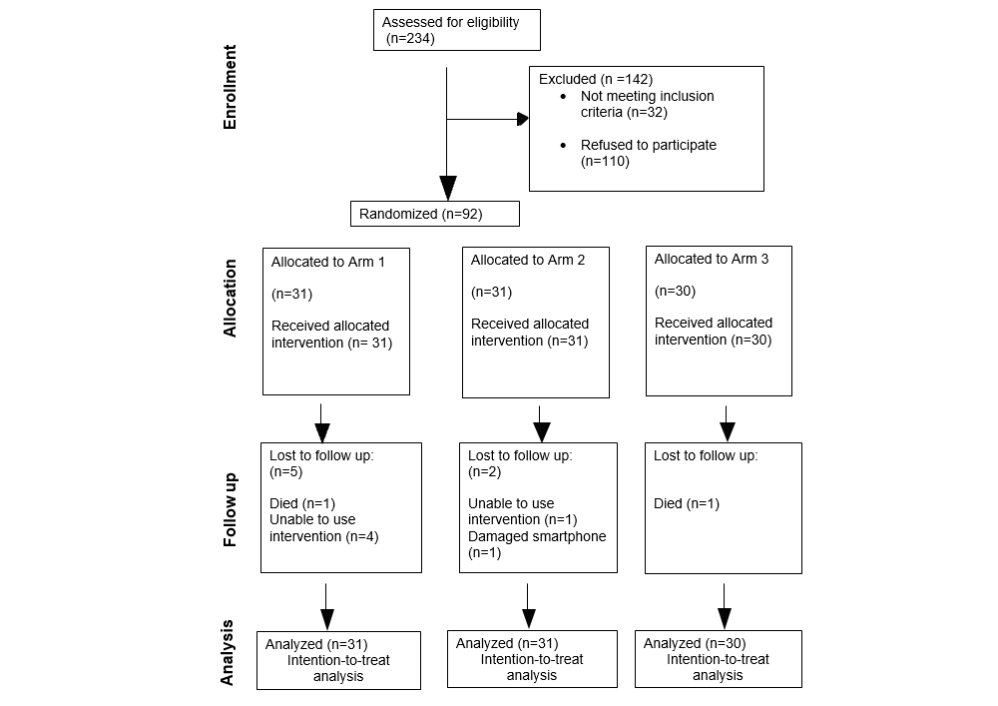
CONSORT flow diagram. ITT: intention-to-treat analysis.

A total of 234 participants were assessed for eligibility from August 2021 to February 2022. From this sample, 202 participants were eligible, and 32 participants were not eligible as they did not meet the inclusion criteria. The main reasons for ineligibility were no smartphone, digital literacy, reduced dexterity, therefore, unable to use the devices, dementia, and blindness. From the sample of 202 eligible participants, 110 participants refused to participate in the trial for various reasons such as “not interested,” “had no time” (n=90), or “ongoing medical investigations” (n=20; see Figure 4 for the CONSORT flow diagram).

A total of 92 participants were randomized to each arm, 31 participants were allocated to arm 1, 31 participants were allocated to arm 2, and 30 participants were allocated to arm 3 (Figure 4). There were 8 participants who were lost to follow-up. Data were collected at the following time points: baseline, 3 (user engagement only), 6, and 12 months. The mean age of participants was 66.8 (SD 7.9) years, ranging from 45 to 81 years of age. There were 57% (52/92) females in the study, with most participants having moderate airway obstruction (43/92, 47%), see Table 1. There were no significant differences in the distribution of baseline characteristics across the three arms except for the variable pulmonary rehabilitation (Table 2). A substantial proportion (84/92, 91%) of the participants reported not engaging with pulmonary rehabilitation at baseline. As this study was conducted during a respiratory pandemic, COVID-19, routine face-to-face pulmonary rehabilitation was not operating due to the Government imposed lockdown restrictions. There was a statistically significant difference, *P*=.01, among the three arms concerning the participants who underwent pulmonary rehabilitation engagement and those who did not (Table 2). Post hoc pairwise comparisons with Bonferroni correction indicated that arm 1 and arm 2 were different from each other with a *P*=.02, with more participants in arm 2 participating in pulmonary rehab in comparison with arm 1. No adverse events were reported during this trial.

**Table 1:** Baseline characteristics of trial participants (N=92).

	Variables		Values	
	**Sex, n (%)**			
		Male	40 (43)	
		Female	52 (57)	
	**Etiology, n (%)**			
		Cigarette smoking	90 (98)	
		Recreational drug abuse	2 (2)	
	**Comorbidities (CCI^a^), n (%)**			
		Mild	56 (61)	
		Moderate	28 (30)	
		Severe	8 (9)	
	**Exercise behavior, n (%)**			
		Engaging	13 (14)	
		Not engaging	78 (85)	
		Other	1 (1)	
	**Nutritional intake, n (%)**			
		Yes	79 (86)	
		No	13 (14)	
	**CAT score^b^** **, n (%)**			
		Low	16 (17)	
		Medium	29 (32)	
		High	35 (38)	
		Very high	12 (13)	
	**Severity, n (%)**			
		Mild	25 (27)	
		Moderate	43 (47)	
		Severe	17 (19)	
		Very severe	7 (7)	
	**Medication, n (%)**			
		No therapy	3 (3)	
		Single therapy	5 (5)	
		Dual therapy	24 (26)	
		Triple therapy	4 (65)	
	Age, mean (SD)		66.8 (7.9)	
	**Flu vaccine, n (%)**			
		Yes	53 (58)	
		No	39 (42)	
	**Pulmonary rehabilitation, n (%)**			
		Yes	8 (9)	
		No	84 (91)	
	**Medication adherence, n (%)**			
		Yes	83 (90)	
		No	7 (8)	
		Not applicable	2 (2)	
	**mMRC^c^** **, n (%)**			
		0	5 (5)	
		1	18 (20)	
		2	44 (48)	
		3	18 (20)	
		4	7 (7)	
	**Quit smoking, n (%)**			
		Yes	45 (49)	
		No	47 (51)	
**Baseline COVID-19 vaccine, n (%)**				
		Yes	89 (97)	
		No	3 (3)	
**COVID-19 vaccine at 6 months, n (%)**				
		Yes	86 (99)	
		No	1 (1)	
**COVID-19 vaccine at 12 months**				
		Yes	84 (99)	
		No	1 (1)	
	**Contracted** **COVID-19 at 6 months, n (%)**			
		Yes	33 (38)	
		No	54 (62)	
	**Contracted** **COVID-19 at 12 months, n (%)**			
		Yes	48 (56)	
		N	37 (44)	

^a^CCI: Charlson Comorbidity Index.

^b^CAT: chronic obstructive pulmonary disease assessment test.

^c^mMRC: modified Medical Research Council Dyspnea Scale.

**Table 2:** Baseline characteristics by the randomized arm.

Variable		Arm 1 (n=31)^a^	Arm 2 (n=31)^b^	Arm 3 (n=30)^c^	*P* value	
**Sex, n (%)**						.63
	Male	15 (48)	14 (45)	11 (37)		
	Female	16 (52)	17 (55)	19 (63)		
**Etiology, n (%)**						.99
	Cigarette smoking	30 (97)	30 (97)	30 (100)		
	Recreational drug abuse	1 (3)	1 (3)	0 (0)		
**Co-morbidities (CCI^d^), n (%)**						.18
	Mild	16 (51)	18 (58)	22 (73)		
	Moderate	12 (39)	9 (29)	7 (23)		
	Severe	3 (10)	4 (13)	1 (3)		
**Exercise behavior, n (%)**						.65
	Engaging	6 (19)	4 (13)	3 (10)		
	Not engaging	25 (81)	26 (84)	27 (90)		
	Other	0 (0)	1 (3)	0 (0)		
**Quit smoking, n (%)**						.51
	Yes	13 (42)	15 (48.4)	17 (57)		
	No	18 (58)	16 (51.6)	13 (43)		
**COVID-19 vaccine, n (%)**						.31
	Yes	31 (100)	30 (97)	28 (93)		
	No	0 (0)	1 (3)	2 (7)		
**Flu vaccine, n (%)**						.81
	Yes	19 (61)	18 (58)	16 (53)		
	No	12 (39)	13 (42)	14 (47)		
**Pulmonary rehabilitation, n (%)**						.01
	Yes	0 (0)	6 (19)	2 (7)		
	No	31 (100)	25 (81)	28 (93)		
**Medicine adherence, n (%)**						.22
	Yes	30 (97)	25 (81)	28 (93)		
	No	1 (3.2)	4 (13)	2 (7)		
	Not applicable	0 (0)	2 (7)	0 (0)		
**Nutritional intake, n (%)**						.86
	Yes	27 (87)	27 (87)	25 (83)		
	No	4 (13)	4 (13)	5 (17)		
**mMRC score^e^** **, n (%)**						.74
	0	2 (6)	2 (6)	1 (3)		
	1	5 (16)	8 (26)	5 (17)		
	2	16 (52)	16 (52)	12 (40)		
	3	6 (19)	4 (13)	8 (27)		
	4	2 (6)	1 (3)	4 (13)		
CAT^f^, mean (SD)		3.0 (1.0)	2.0 (1.5)	3.0 (1.0)	.35	
**Medication** **, n (%)**						.06
	No therapy	0 (0)	3 (10)	0 (0)		
	Single therapy	4 (13)	0 (0)	1 (3)		
	Dual therapy	10 (32)	8 (26)	6 (20)		
	Triple therapy	17 (55)	20 (64)	23 (77)		
Severity, mean (SD)		2.0 (1.0)	2.0 (1.0)	2.0 (2.0)	.58	
Age (years), mean (SD)		65.7 (7.7)	66.5 (8.5)	68.2 (7.3)	.45	

^a^Arm 1 includes a self-management app, monthly phone calls, and standard respiratory outpatient care.

^b^Arm 2 is the self-management app and standard respiratory outpatient care.

^c^Arm 3 is standard respiratory outpatient care, the control group.

^d^CCI: Charlson Comorbidity Index.

^e^mMRC: modified Medical Research Council Dyspnea Scale.

^f^CAT: chronic obstructive pulmonary disease.

### COPD Exacerbations Reviewed in the GP and Hospital Settings

**Figure 5:**
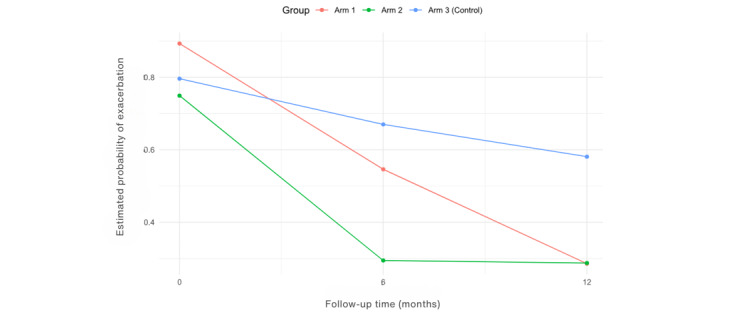
Estimated probabilities of an exacerbation over time in the GP setting.

**Figure 6:**
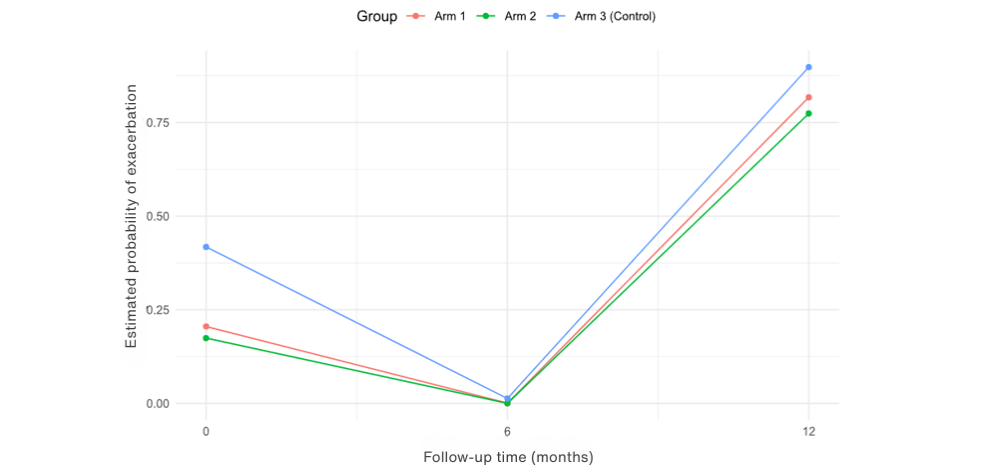
Estimated probabilities of an exacerbation over time in the hospital setting.

The proportion of participants in each arm that experienced exacerbations in the hospital and GP setting is reported in Table 3. There was a statistically significant difference in the proportion of participants who had a COPD exacerbation versus those who did not have an exacerbation at 6 months in the hospital setting across the three arms, *P*=.03 (Table 3). Post hoc pairwise comparisons (*P*=.02) revealed that intervention arm 2 experienced fewer exacerbations reviewed in the hospital setting than those in the control arm. The intervention arms had significantly lower odds of developing a COPD exacerbation in both the GP (with an adjusted OR of 0.20 (95% CI 0.06-0.61; *P*=.005) and hospital (OR 0.26, 95% CI 0.07-0.99; *P*=.049 and OR 0.18, 95% CI 0.04-0.78, *P*=.02) setting compared with the control arm (Table 4). The probability of developing a COPD exacerbation for participants with severe severity is 6.55 times significantly higher than for participants with moderate severity, with *P*=.009 (Table 4). There was a statistically significant difference (*P*=.01) at 6 months in the GP setting, indicating that the intervention arm had a lower risk of developing an exacerbation than the control arm (Table 5). The control arm had the highest estimated probability of having a COPD exacerbation over time, see Figure 5. At 6 months, in the hospital setting, there was a statistically significant difference (*P*=.01 and *P*=.006), indicating the intervention arms had a significantly lower risk of developing a COPD exacerbation compared with the control arm (Table 6). However, after 6 months of follow-up, there was a sharp increase in all the arms, with the control arm having the highest and the intervention arms having the lowest estimated probabilities of developing an exacerbation (Figure 6). The risk of developing an exacerbation was 0.04 times lower for participants with mild severity compared with participants with severe severity, with a statistically significant (*P*=.02; Table 6). Sensitivity analysis indicated a significant difference at 12 months (*P*=.02), where the intervention arms experienced fewer exacerbations than the control arm at 12 months (see Multimedia Appendix 3). Participants with missing data in the control arm were assumed to have experienced an exacerbation (negative outcome), while participants with missing data in the intervention arms (arms 1 and 2) were considered to have experienced no exacerbation (best outcome). However, these results may not accurately reflect the true outcomes of participants with missing data, leading to differences in statistical significance.

**Table 3:** Comparison of proportions of participants with and without an exacerbation in the general practitioner (GP) and hospital settings across arms.

Variable			Arm 1 (n=31)	Arm 2 (n=31)	Arm 3 (n=30)	*P* value	
**Hospital**							
	**Baseline**						.08
		Yes, n (%)	10 (32)	10 (32)	17 (57)		
		No, n (%)	21 (68)	21 (68)	13 (43)		
		Missing, n	0	0	0		
	**6 months**						.03
		Yes, n (%)	3 (11)	2 (6)	9 (31)		
		No, n (%)	24 (89)	29 (94)	20 (69)		
		Missing, n	4	0	1		
	**12 months**						.88
		Yes, n (%)	5 (19)	6 (21)	7 (24)		
		No, n (%)	21 (81)	23 (79)	22 (76)		
		Missing, n	5	2	1		
**General practitioner**							
	**Baseline**						.50
		Yes, n (%)	28 (90)	25 (81)	24 (80)		
		No, n (%)	3 (10)	6 (19)	6 (20)		
		Missing, n	0	0	0		
	**6 months**						.10
		Yes, n (%)	12 (44)	8 (26)	15 (52)		
		No, n (%)	15 (56)	23 (74)	14 (48)		
		Missing, n	4	0	1		
	**12 months**						.10
		Yes, n (%)	11 (42)	11 (38)	19 (66)		
		No, n (%)	15 (58)	18 (62)	10 (34)		
		Missing, n	5	2	1		

**Table 4:** Generalized linear mixed model (GLMM) results for general practitioner (GP) and hospital settings.

Variable			General practitioner				Hospital		
			Unadjusted, OR^a^ (95% CI)	Adjusted, OR (95% CI)	*P* value	Unadjusted, OR (95% CI)		Adjusted, OR (95% CI)	*P* value
Arm 1^b^			0.45 (0.15-1.34)	0.41 (0.14-1.22)	.11	0.37 (0.09-1.52)		0.26 (0.07-0.99)	.04
Arm 2^c^			0.25 (0.08-0.76)	0.20 (0.06-0.61)	.005	0.28 (0.07-1.20)		0.18 (0.04-0.78)	.02
**Severity**									
	Moderate		3.70 (1.15-11.86)	4.28 (1.37-13.34)	.01	5.54 (1.00-30.61)		—^d^	—
	Severe		5.28 (1.26-22.11)	6.55 (1.60-26.90)	.009	5.68 (0.79-40.99)		—	—
	Very Severe		3.79 (0.63-22.70)	2.79 (0.51-15.38)	.23	9.27 (0.84-101.95)		—	—
**Comorbidities**									
		Mild	4.97 (0.58-42.97)	—	—	0.24 (0.03-2.12)		0.10 (0.01-0.98)	.048
		Moderate	4.65 (0.50-43.20)	—	—	1.04 (0.12-9.29)		0.58 (0.07-4.77)	.60
		Baseline smoking	1.54 (0.64-3.71)	—	—	1.31 (0.42-4.04)		—	—
		Age	0.99 (0.93-1.04)	—	—	1.05 (0.97-1.14)		—	—
		Gender	0.83 (0.35-2.02)	—	—	2.06 (0.63-6.75)		—	—
		Time	1.69 (0.82-3.46)	—	—	1.59 (0.64-3.95)		—	—

^a^OR: odds ratio.

^b^Arm 1 includes a self-management app, monthly phone calls, and standard respiratory outpatient care.

^c^Arm 2 is the self-management app and standard respiratory outpatient care.

^d^Not applicable.

**Table 5:** Logistic model results for general practitioner (GP) setting.

Variable	Baseline			6 months			12 months	
	OR (95% CI)	*P* value	OR (95% CI)		*P* value	OR (95% CI)		*P* value
Arm 1^a^	2.14 (0.44-12.28)	.35	0.59 (0.16-2.02)		.40	0.29 (0.08-0.96)		.04
Arm 2^b^	0.77 (0.17-3.29)	.71	0.21 (0.05-0.71)		.01	0.29 (0.08-0.95)		.04
Comorbidities: mild	1.55 (0.17-10.54)	.66	1.76 (0.16-43.09)		.66	3.56 (0.38-81.90)		.31
Comorbidities: moderate	2.59 (0.24-24.63)	.40	1.99 (0.18-49.12)		.60	1.98 (0.19-46.42)		.59
Severity: mild	2.59 (0.37-17.87)	.32	0.24 (0.03-1.85)		.16	0.44 (0.06-2.83)		.38
Severity: moderate	5.98 (0.83-43.99)	.06	2.10 (0.36-13.83)		.41	1.02 (0.16-6.19)		.98
Severity: severe	16.26 (1.38-433.05)	.04	3.27 (0.47-26.91)		.24	1.00 (0.13-7.09)		.99
Age	0.97 (0.88-1.06)	.49	0.98 (0.91-1.05)		.55	1.01 (0.94-1.08)		.75
Smoking status: yes	0.98 (0.25-3.74)	.97	1.30 (0.44-3.97)		.63	0.33 (0.11-0.95)		.04
Sex: female	1.97 (0.56-7.34)	.29	1.00 (0.35-2.83)		.99	0.39 (0.13-1.06)		.07

^a^Arm 1 includes a self-management app, monthly phone calls, and standard respiratory outpatient care.

^b^Arm 2 is the self-management app and standard respiratory outpatient care.

**Table 6:** Logistic model results for the hospital setting.

Variable	Baseline		6 months			12 months	
	OR (95% CI)	*P* value	OR (95% CI)	*P* value	OR (95% CI)		*P* value
Arm 1^a^	0.36 (0.11-1.09)	.07	0.06 (0.0046-0.45)	.01	0.51 (0.09-2.37)		.39
Arm 2^b^	0.29 (0.09-0.89)	.03	0.02 (0.001-0.24)	.006	0.39 (0.08-1.76)		.23
Comorbidities: mild	0.85 (0.15-5.30)	.85	0.04 (0.001-1.84)	.08	0.06 (0.004-0.80)		.03
Comorbidities: moderate	0.79 (0.13-5.29)	.80	0.66 (0.03-22.11)	.79	0.28 (0.03-3.23)		.28
Severity: mild	0.62 (0.09-4.19)	.61	0.26 (0.01-8.12)	.38	0.04 (0.001-0.49)		.02
Severity: moderate	1.29 (0.22-8.25)	.77	1.79 (0.18-42.16)	.64	0.26 (0.04-1.79)		.16
Severity: severe	2.64 (0.39-19.99)	.32	0.99 (0.06-27.47)	.99	0.74 (0.10-5.69)		.76
Age	1.01 (0.95-1.08)	.69	1.09 (0.97-1.25)	.16	0.99 (0.91-1.08)		.88
Smoking: yes	1.01 (0.38-2.67)	.98	0.44 (0.08-2.05)	.30	0.97 (0.26-3.61)		.96
Sex: female	0.72 (0.28-1.82)	.48	2.59 (0.52-16.77)	.26	3.38 (0.89-1.56)		.09

^a^Arm 1 includes a self-management app, monthly phone calls, and standard respiratory outpatient care.

^b^Arm 2 is the self-management app and standard respiratory outpatient care.

### Engagement

**Figure 7:**
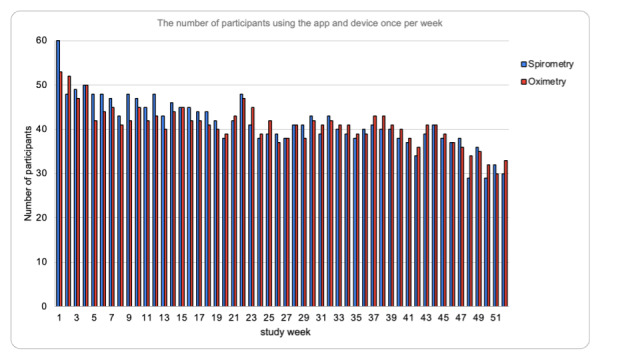
App logins and use of devices over 12 months.

There was a statistically significant difference in the distribution of the self-reported user engagement scale across the intervention arms at 6 months and 12 months of follow-up (*P*=.02 and *P*=.03, respectively; Table 7). Arm 1 reported better engagement than arm 2 at 6 and 12 months. The Friedman rank sum test was used to analyze the potential differences in engagement using the app across the three time points. The test yielded a Friedman chi-squared statistic of 1.273 (*P*=.52). There were no statistically significant differences in the median of user app engagement across the three time points, which indicates that the monthly phone calls in arm 1 did not have a statistically significant effect on engagement at 3, 6, and 12 months of follow-up. Engagement was sustained across the 12 months among participants in this trial. The average adherence among participants using the pulse oximeter was 66.8 (SD 33.5) and 51.7 (SD 31.7) using the spirometry device over 52 weeks (Table 8). App usage determined by app logins was stable among the intervention arms from week 1 to week 46. Following week 46 until week 52, there was a gradual decline in using the app and devices noted among 40% (n=22) of participants in the intervention arms (Figure 7). Participants in the intervention arms were sent a letter 6 weeks in advance of the 12-month appointment informing them that the trial was due to end. This may have contributed to the reduced engagement from week 46 until 52, as participants were aware the trial was coming to an end. However, participants engaged with the app once or more times a week despite age, gender, or severity of disease (Figure 7). One participant withdrew from this study due to unresolved technical issues.

**Table 7:** User engagement for the three follow-up periods.

Variable		Arm 1 (n=31)^a^, n (%)	Arm 2 (n=31)^b^, n (%)	*P* value	
**3 months**					.60
	Average engagement	4 (13.3)	3 (9.7)		
	Very good	21 (70.0)	26 (83.9)		
	Excellent	5 (16.7)	2 (6.5)		
**6 months**					.02
	Average engagement	2 (7.4)	8 (25.8)		
	Very good	19 (70.4)	21 (67.7)		
	Excellent	6 (22.2)	2 (6.5)		
**12 months**					.04
	Average engagement	1 (3.9)	5 (16.7)		
	Very good	18 (69.2)	22 (73.3)		
	Excellent	7 (26.9)	3 (10)		

^a^Arm 1 includes a self-management app, monthly phone calls, and standard respiratory outpatient care.

^b^Arm 2 is the self-management app and standard respiratory outpatient care.

**Table 8:** Adherence to using devices.

Variable	Arm 1^a^, mean (SD)	Arm 2^b^, mean (SD)	Arm 3^c^, mean (SD)	*P* value
Adherence to the pulse oximeter	67.0 (33.8)	66.7 (33.7)	—^d^	.97
Adherence with spirometry	52.1 (34.6)	51.3 (29.1)	—	.91

^a^Arm 1 includes a self-management app, monthly phone calls, and standard respiratory outpatient care.

^b^Arm 2 is the self-management app and standard respiratory outpatient care.

^c^Arm 3 is the standard respiratory outpatient care, the control group.

^d^Not applicable.

### Physical Activity

Most participants (n=84, 91%) reported not engaging with pulmonary rehabilitation at baseline (Table 1). There was a statistically significant difference (*P*=.01) among the 3 arms concerning the participants who participated in pulmonary rehabilitation and those who did not (Table 2). Post hoc pairwise comparisons with Bonferroni correction indicated that arm 1 and arm 2 were different from each other (*P*=.02) with more participants in arm 2 participating in pulmonary rehab in comparison to arm 1. Exercise capacity at both 6 and 12 months of follow-up was statistically significant (*P*=.02 and *P*=.03, respectively; Table 9). Post hoc analysis revealed that the intervention arm 1 illustrated an improved exercise capacity than the control group (*P*=.03).

Also, there was a statistically significant difference in the mean step count (*P*=.009; Table 9). Post hoc pairwise comparisons with Bonferroni correction revealed that arm 2 had an increased step count in comparison to the control group (*P*=.01).

**Table 9:** Summary statistics for secondary outcomes in randomized groups.

Variable				Arm 1^a^		Arm 2^b^		Arm 3^c^		*P* value	
**Baseline**											
	Exercise capacity, mean (SD)			2.0 (1.0)		3.0 (2.0)		3.0 (2.0)		.24	
	HRQoL^d^, mean (SD)			3.0 (1.0)		3.0 (1.0)		3.0 (1.0)		.98	
	Self-efficacy, mean (SD)			3.0 (1.5)		3.0 (2.0)		3.0 (2.0)		.71	
	Step count, mean (SD)			5250 (5764)		3500 (4253)		3500 (3000)		.18	
	mMRC score^e^, n (%)										.74
		0	2 (6)		2 (6)		1 (3)				
		1	5 (16)		8 (26)		5 (17)				
		2	16 (52)		16 (52)		12 (40)				
		3	6 (19)		4 (13)		8 (27)				
		4	2 (6)		1 (3)		4 (13)				
	CAT score^f^, mean (SD)			3.0 (1.0)		2.0 (1.5)		3.0 (1.0)		.35	
**6 months**											
	Exercise capacity, mean (SD)			2.0 (1.0)		2.0 (1.0)		3.0 (2.0)		.02	
	HRQoL, mean (SD)			2.0 (1.0)		2.0 (1.0)		2.0 (1.0)		.71	
	Self-efficacy, mean (SD)			4.0 (1.5)		3.0 (1.5)		3.0 (2.0)		.97	
	Step count, mean (SD)			4650 (6863.3)		3900 (4100)		3500 (6000)		.28	
	mMRC score, n (%)										.77
		0	4 (15)		5 (16)		1 (3)				
		1	10 (37)		11 (35)		12 (41)				
		2	7 (26)		9 (29)		7 (24)				
		3	4 (15)		5 (16)		5 (17)				
		4	2 (7)		1 (3)		4 (14)				
	CAT score, mean (SD)			2.0 (2.0)		2.0 (0.5)		2.0 (1.0)		.65	
**12 months**											
	Exercise capacity, mean (SD)			2.0 (1.0)		2.0 (1.0)		3.0 (1.0)		.03	
	HRQoL, mean (SD)			2.0 (0.75)		2.0 (0.0)		2.0 (1.0)		.16	
	Self-efficacy, mean (SD)			4.0 (2.0)		4.0 (2.0)		3.0 (1.0)		.54	
	Step count, mean (SD)			4500 (6001)		4800 (6315.5)		999 (3251)		.009	
	mMRC score, n (%)									.14	
		0	2 (8)		1 (3)		3 (10)				
		1	11 (42)		15 (50)		6 (21)				
		2	8 (31)		6 (20)		7 (24)				
		3	2 (8)		7 (23)		10 (34)				
		4	3 (12)		1 (3)		3 (10)				
	CAT, mean (SD)			2.0 (1.0)		2.0 (1.0)		2 (1.0)		.08	

^a^Arm 1 includes a self-management app, monthly phone calls, and standard respiratory outpatient care.

^b^Arm 2 is the self-management app and standard respiratory outpatient care.

^c^Arm 3 is the standard respiratory outpatient care, the control group.

^d^HRQoL: health-related quality of life.

^e^mMRC: modified Medical Research Council Dyspnea Scale.

^f^CAT: chronic obstructive pulmonary disease assessment test.

### Assessment of HRQoL

Participants with poor control of HRQoL decreased from 53 participants at baseline to 29 and 20 participants at 6 and 12 months, respectively (Multimedia Appendix 4 shows the summary statistics for secondary outcomes). Although there was no statistically significant difference in terms of HRQoL across the 3 arms during the various time points, participants in the intervention arms were at a lower risk of developing an exacerbation in comparison to the control arm, which impacts HRQoL. Furthermore, in the intervention arms, the relative risk of 0.55 (95% CI 0.30-0.99) and 0.45 (95% CI 0.24-0.85) at baseline signified a 45% significantly lower risk of developing a high CAT score indicating uncontrolled and symptomatic COPD in comparison to the control arm (Table 10).

**Table 10:** Relative risk (RR), absolute risk reduction (ARR), and 95% CI for the secondary outcomes.

Variables and arms			Baseline	6 months	12 months
**RR^a^** **(95% CI)**					
	**Exercise capacity**				
		Arm 1	0.45 (0.24-0.85)	0.33 (0.16-0.71)	0.35 (0.16-0.74)
		Arm 2	0.52 (0.28-0.94)	0.42 (0.22-0.80)	0.40 (0.20-0.78)
	**HRQoL^b^**				
		Arm1	0.68 (0.39-1.18)	0.44 (0.23-0.88)	0.27 (0.12-0.62)
		Arm 2	0.71 (0.41-1.22)	0.32 (0.16-0.66)	0.23 (0.10-0.53)
	**Self-efficacy**				
		Arm 1	0.61 (0.35-1.08)	0.44 (0.23-0.87)	0.42 (0.21-0.86)
		Arm 2	0.58 (0.32-1.04)	0.52 (0.28-0.94)	0.41 (0.21-0.81)
	**CAT^c^** **score**				
		Arm 1	0.55 (0.30-0.99)	0.30 (0.13-0.65)	0.23 (0.09-0.56)
		Arm 2	0.45 (0.24-0.85)	0.19 (0.08-0.46)	0.23 (0.10-0.53)
	**mMRC^d^** **score**				
		Arm 1	0.26 (0.12-0.56)	0.22 (0.09-0.54)	0.19 (0.07-0.50)
		Arm 2	0.16 (0.06-0.41)	0.19 (0.08-0.46)	0.27 (0.12-0.58)
**ARR^e^** **(95% CI)**					
	**Exercise capacity**				
		Arm 1	–0.25 (–0.42 to –0.07)	–0.32 (–0.50 to –0.14)	–0.34 (–0.53 to –0.16
		Arm 2	–0.18 (–0.36 to –0.01)	–0.24 (–0.41 to –0.06)	–0.29 (–0.46 to -0.11)
	**HRQoL**				
		Arm 1	–0.02 (–0.19 to 0.14)	0.09 (–0.09 to 0.29)	–0.14 (–0.32 to 0.03)
		Arm 2	0.01 (–0.15 to 0.17)	–0.02 (–0.19 to 0.14)	–0.18 (–0.33 to -0.03)
	**Self-efficacy**				
		Arm 1	–0.09 (–0.26 to 0.084)	–0.07 (–0.26 to 0.11)	–0.09 (–0.28 to 0.10)
		Arm 2	–0.12 (–0.29 to 0.05)	–0.001 (–0.18 to 0.17)	–0.10 (–0.28 to 0.08)
	**CAT score**				
		Arm 1	0.02 (–0.16 to 0.19)	–0.05 (–0.22 to 0.12)	–0.18 (–0.34 to –0.02)
		Arm 2	–0.08 (–0.26 to 0.09)	–0.15 (–0.29 to –0.01)	–0.18 (–0.33 to –0.03)
	**mMRC score**				
		Arm 1	–0.14 (–0.30 to 0.01)	–0.09 (–0.24 to 0.07)	–0.26 (–0.41 to –0.10)
		Arm 2	–0.24 (–0.37 to –0.11)	–0.12 (–0.26 to 0.02)	0.18 (–0.34 to –0.02)

^a^RR: relative risk.

^b^HRQoL: health-related quality of life.

^c^CAT: chronic obstructive pulmonary disease assessment test.

^d^mMRC: modified Medical Research Council Dyspnea Scale.

^e^ARR: absolute risk reduction.

### Breathlessness

The calculated relative risk relating to the mMRC within the intervention arms was 0.26 (95% CI 0.12-0.56) and 0.16 (95% CI 0.06-0.41). This finding suggests that participants in the intervention arms had a substantially lower significant risk of experiencing high levels of breathlessness compared with those in the control arm at baseline, 6 months, and 12 months (Table 10).

### Self-Efficacy

There was no statistically significant difference across the three arms during the various time points relating to self-efficacy (Table 9). However, it was evident that the intervention arms had a lower risk of poor self-efficacy at 6 and 12 months compared with the control arms (Table 10).

## Discussion

### Principal Findings

This pilot RCT explored the effect of a smartphone app self-management program on clinical health outcomes among COPD participants over 12 months. This study demonstrated statistical significance in the intervention arms whereby there were fewer exacerbations presenting in the hospital setting and improved exercise capacity and step count in comparison with the standard respiratory outpatient care.

It was apparent that there were significantly fewer exacerbations (*P*=.03) at 6 months in the hospital setting in comparison with the control arm. Furthermore, the odds of developing an exacerbation were lower in the intervention arms in comparison to the control arm. In this trial, participants in the intervention arms had access to educational videos through the app pertaining to recognizing symptoms of an exacerbation and how to self-manage their symptoms or seek help early. In addition, these videos provided education related to the importance of medication adherence and guidance on how to engage with a self-management plan. The main difference between the arms was the use of a smartphone app self-management program, which perhaps resulted in increased knowledge, awareness, and confidence, empowering participants to self-manage their chronic illness, resulting in reduced COPD exacerbation rates and hospitalizations among the intervention arms. This finding was similar in other studies [17,18,22]. Reducing exacerbation rates reduces hospitalizations and morbidities and prevents premature death among this cohort [1,16,44-48]. Also, reducing hospital admissions among this cohort may demonstrate cost effectiveness resulting in reducing the economic burden for healthcare providers [10-12].

There was an increase noted in the estimated probability of developing an exacerbation after 6 months in the hospital setting, which may have resulted in 48% (n=56) of participants reporting contracting COVID-19 at the 12-month follow-up. This may have resulted in increased hospital visits. Also, the 12 months of follow-up included the winter season in Ireland, resulting in high rates of influenza, COVID-19, and pneumonia that would have impacted participants in this trial who were susceptible to these respiratory illnesses due to their underlying respiratory condition. However, the control arm had the highest probability of developing a COPD exacerbation following 6 months in the hospital setting. The findings from this trial illustrated that the risk of developing a COPD exacerbation was 0.04 times lower for participants with a mild level of disease compared to participants with a higher severity of disease, and this is congruent with the literature [1-3]. The majority of participants (73%, 67/92) in this trial had moderate to severe COPD, resulting in a higher proportion of participants at risk of developing an exacerbation in comparison to those with mild severity (n=25). As this study was conducted during the COVID-19 pandemic, routine face-to-face pulmonary rehabilitation was not operating due to the Government-imposed lockdown restrictions. The main benefits of pulmonary rehabilitation include reduced exacerbations resulting in fewer hospitalizations, enhanced physical activity, improved respiratory symptoms, and quality of life [1,2,9,14]. Most participants in this study did not engage in pulmonary rehabilitation at baseline, which may have also affected the exacerbation rate in the GP and hospital setting.

There was a statistically significant improvement in exercise capacity at both 6 and 12 months in the intervention arm in comparison with the control arm (*P*=.02 and *P*=.03). Also, the intervention arm demonstrated improved physical activity in the form of step count in comparison to the control arm (*P*=.009). The reason for this may be that participants in the intervention arms were encouraged to monitor and track goals in relation to their step count. In addition, they had access to educational videos that emphasized the importance of physical activity in maintaining self-control of their chronic illness, COPD. Previous studies have highlighted that including physical activity in a self-management program improves their exercise capacity, which positively affects other clinical health outcomes such as HRQoL, symptom control, and self-efficacy in patients with COPD [13-16,20].

All participants in this study engaged with the app despite age, gender, or severity of disease, and this was similar to other studies [17,25,26]. Interestingly, the monthly phone calls in arm 1 did not have a statistically significant effect on app engagement during this study. This suggests that factors other than the frequency of phone calls contributed to the observed changes in engagement levels over time. Technical issues did not cause harm but were inconvenient to participants, which may have resulted in reduced engagement. Technical issues such as loss of functionality of the app, inability to pair devices with Bluetooth on the smartphone, duplicate recordings, and updating and advancing the app occurred during the study. This is a common problem when using smartphone apps and has been frequently reported in the literature [12,15,16,23-28]. Given that technology is advancing rapidly [49], it is envisioned that smartphone apps will become a natural complement within clinical practice [29,50,51]. Therefore, there is a need for investment in technical advances and support to be readily available for participants to support engagement levels using self-management apps. Previous studies reported sustained engagement for durations of 3 months [17], 8 weeks [26], and 6 weeks [24,25]; however, they noted a gradual decline toward the end of the study [17,24-26]. In this trial, there was a gradual decline in using the app and devices noted among 40% (n=22/55) of participants in the intervention arms, which was unrelated to technical issues. Participants in the intervention arms were sent a letter 6 weeks in advance of the 12-month appointment informing them that the trial was due to end. This may have contributed to the reduced engagement from week 46 until 52 as participants were aware that the trial was coming to an end, the Hawthorne effect commonly reported in RCTs. Despite this, 60% (33/55) of participants in the intervention arms continued to use the app and devices for 12 months. As outlined in previous studies [17,18,22,52], perhaps participants in this study were motivated to engage with the app as they experienced benefits such as reduced hospital visits due to an exacerbation, improved physical activity, knowledge, awareness, and confidence in self-managing their chronic illness. Furthermore, as noted in the literature, engaging with self-management apps empowers participants to discuss aspects of their chronic illness at their next health care appointment, thereby enhancing the rapport between patients and health care practitioners [12,53].

### Strengths and Limitations

The strength of this trial was the use of a comprehensive self-management program comprised of monthly education, symptom tracking, goal setting, communication with a health care professional, and weekly motivational messages. However, smartphone technology investigated in previous studies among participants with COPD only focused on certain aspects of a self-management program, resulting in issues with heterogeneity among interventions used [15-28]. A self-management program needs to address a broader number of relevant issues in order to increase the user’s knowledge, confidence, and skills to self-manage their chronic illness [1,47]. This is the first national pilot RCT exploring the effect of a smartphone app self-management program on clinical health outcomes in adults with COPD over 12 months. Only a few international longitudinal studies have been published on this topic [18,20]. Therefore, this study will contribute to the body of existing knowledge on smartphone apps supporting a self-management program among individuals with COPD. The results of this trial demonstrated significant benefits in clinical health outcomes, such as reduced exacerbation rates in the hospital setting, improved exercise capacity, and increased step count among the intervention arms in comparison with standard care. These benefits may have occurred due to readily available education on their smartphone pertaining to self-management, symptom tracking, motivation, and goal setting that resulted in enhanced self-monitoring skills among participants in the intervention arms. Finally, this study will inform a larger trial in terms of methodology, eligibility criteria, recruitment process, sample size, randomization procedures, data collection, and data analysis process. Furthermore, this trial will allow for a review of case definitions of primary outcomes, an overview of retention rates among trial participants, and unanticipated challenges that occurred, which can be addressed when preparing for a larger trial.

This was a single-center trial, which is limiting. Although the sample obtained was from a large academic teaching hospital in the Republic of Ireland, and the findings may be reflective of sites of similar context, they are not generalizable to all hospital settings. In addition, this trial is limited by design and, due to the small sample size, lacks adequate power to demonstrate significant effects on all measured outcomes. This study made multiple comparisons between arms, which can lead to a false inference. However, this was a pilot study, and the results will not be used to make treatment decisions but can be used to generate hypotheses in subsequent, adequately powered trials. This study will provide valuable data for expanding to a multicenter trial. Another limitation is that technical issues experienced during the trial may have limited engagement levels among participants. This is a common problem when using smartphone apps and has been frequently reported in the literature [12,15-28]. There is a need for investment in technical advances and support to be readily available for participants to support engagement levels using self-management apps.

### Future Research

It was evident from this trial, similar to the literature, that self-management apps via a smartphone only appeal to a certain cohort of participants for many reasons, such as digital literacy, not owning a smartphone, lack of internet connectivity, poor dexterity to use a smartphone or simply not interested in self-managing their COPD [16-20,22,27,28,35]. It is worth noting that this trial and other studies were conducted before and during the COVID-19 pandemic [16-20,22-28]. Since the COVID-19 pandemic, the use of smartphone apps has increased the delivery of health care both nationally and internationally [29,30]. Given the increased use of digital technology in the delivery of health care currently, further research is required to ascertain if the refusal rate to participate in similar studies remains high. In addition, larger future studies could explore the impact of monthly phone calls relating to the content of the phone calls, user preferences, or external circumstances that may influence app engagement levels among this population. Finally, there is a need for a larger multicenter trial exploring the effect of a smartphone app self-management program on clinical health outcomes in adults with COPD.

### Conclusion

This study demonstrated that a smartphone app self-management program had a positive effect on clinical health outcomes for participants with COPD in comparison to standard care. Furthermore, this study demonstrated statistical significance in the intervention arms whereby there were fewer exacerbations presenting in the hospital setting and improved exercise capacity and physical activity in comparison to the standard respiratory outpatient care. The majority of participants engaged with the app for 12 months. There is a need for more longitudinal multicenter studies with fully powered samples to confirm sustained benefits and perhaps identify more statistically significant events.

## Appendix

Multimedia Appendix 1 CONSORT-eHEALTH checklist (V 1.6.1).

Multimedia Appendix 2 Intervention study pack.

Multimedia Appendix 3 Sensitivity analysis.

Multimedia Appendix 4 Summary statistics for secondary outcomes.

## Abbreviations

CAT: chronic obstructive pulmonary disease assessment test
CONSORT: Consolidated Standards of Reporting Trials
COPD: chronic obstructive pulmonary disease
FEV1: forced expiratory volume at 1 second
GLMM: generalized linear mixed model
GP: general practitioner
HRQoL: health-related quality of life
ITT: intention-to-treat analysis
mMRC: modified Medical Research Council Dyspnea Scale
OPD: outpatient department
OR: odds ratio
pMp: PatientMpower
RCT: randomized controlled trial
SpO2: peripheral oxygen saturation

## Data Availability

The datasets generated or analyzed during this study are available from the corresponding author on reasonable request. All relevant data are included in this article.
