# Supplementary material for: A Smartphone App Self-Management Program for Chronic Obstructive Pulmonary Disease: Randomized Controlled Trial of Clinical Outcomes
Source: JMIR Mhealth Uhealth. 2025 Apr 23;13:e56318. doi: 10.2196/56318 (PMC12059498; doi:10.2196/56318)
Supplement: Multimedia Appendix 2 [file mhealth_v13i1e56318_app2.docx]

# Intervention study pack

The research team and patientMpower (pMp, company supplying the app and devices) will help you to download and set up the self-management programme app.

- Firstly, you will receive an email informing you that you are now ready to download and set up the patientMpower App.
- Click on the first link which will open in your web browser and direct you to setting up your password. If the link doesn’t work, you can copy and paste the web address provided in the email into your web browser and press search.
- Set up your password. Remember your password must have the following;
- At least 8 characters in length
- Contain one uppercase letter
- Contain one lowercase letter
- Contain one number
- Once the password has been set, return to the email, and select the link to download the app from either the Apple Store or the Google Play Store on your smartphone.
- Login into the app using the email and password you have created.
- You do not need to sign up to the app (Figure 1).
- Read the consent policy and terms and conditions.

**Figure 1:**


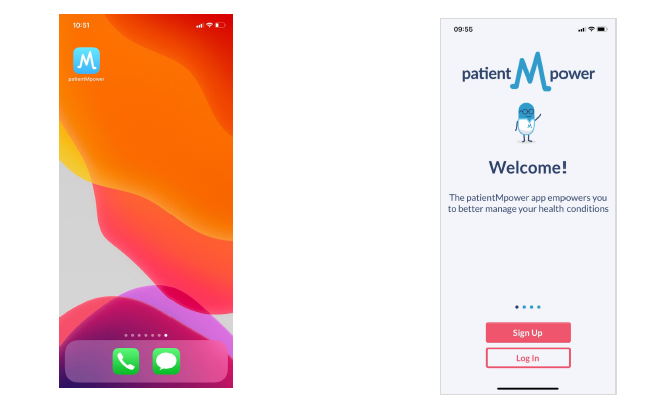


**Setting up the spirometer**


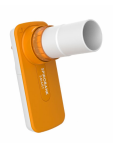


1. Open the box and remove the device and 2 AAA batteries

2. Slide down to open the spirometer battery door

3. Insert both batteries

4. Slide the battery door back on

**Setting up the pulse oximeter**


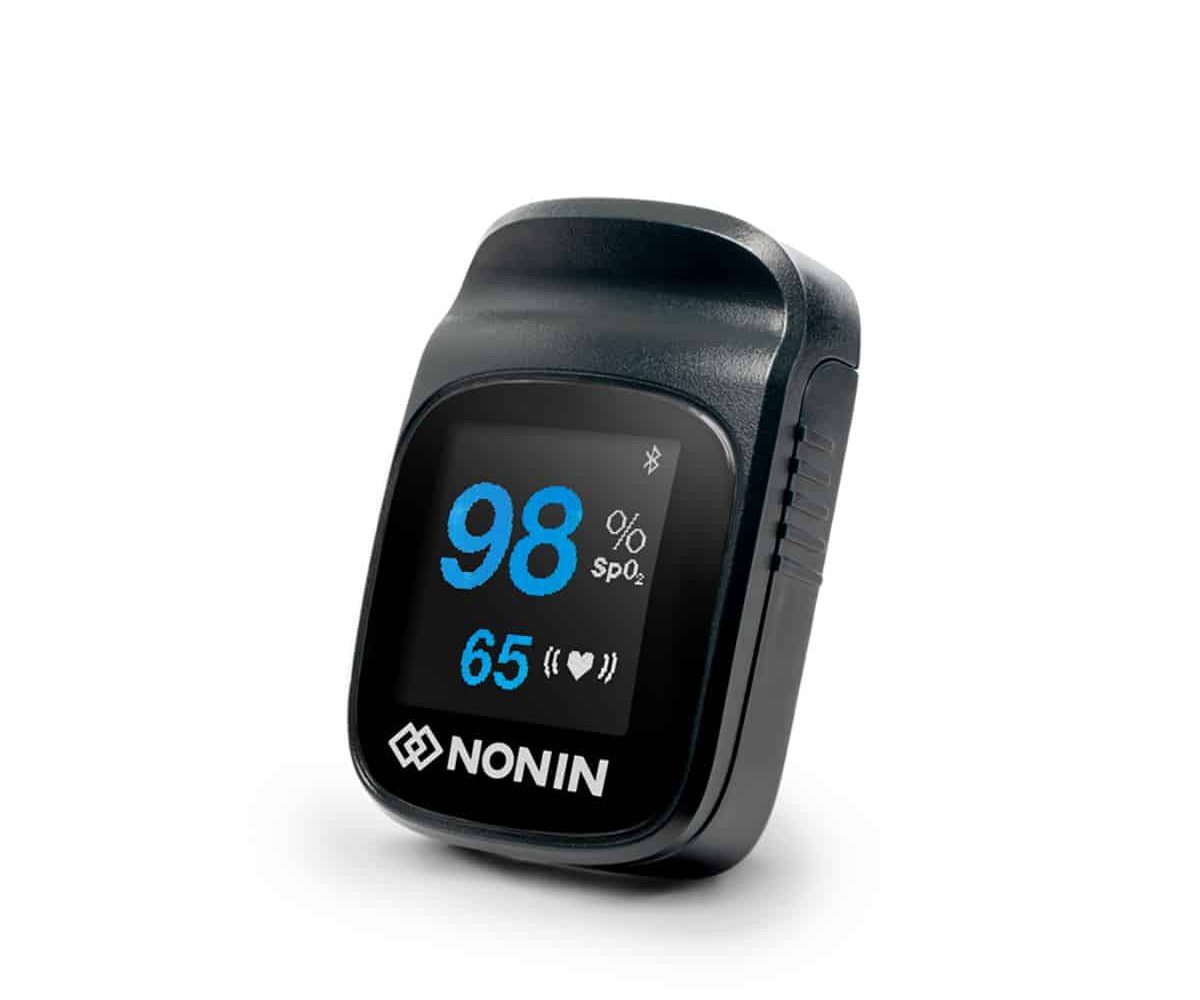


1) Open the box and remove the device two AAA batteries which are also provided inside the box

2) Slide down to open the pulse oximeter battery door

3) Insert both batteries. Note that on the left-hand side the positive end of the battery is at the bottom and this is opposite for the right-hand side (positive end is at the top).

4) Slide the battery door back on.

**Connecting the Spirobank Spirometer/ Pulse Oximeter to your phone**

1. Turn on bluetooth on your phone.

2. Select “More” and “Bluetooth Devices”.

3. Allow the app to access your location while using the app (Android phones only).

4. You should see the device appear SPIROBANK SMART spirometer click this and press pair in the pop up.

5. Once paired you will see a success message.

6. To connect the pulse oximeter, place the device on your finger until you see Nonin 3230 appear on the phone screen. Click this and press pair in the pop up.

**Using the Spirometer**

Remember prior and during using the spirometer;

- Try not to cough
- Maintain good posture
- Breath out hard for at least 6 seconds

1. Press the add measurement button and select “Add Spirometry”

2. Follow the instructions on screen to take the measurement. Following this a video with audio and visual content would play describing how to complete the measurement using the device.

3. Take 2-3 normal breaths and then one slow deep breath, then blow as hard and fast as you can keep going until you completely empty your lungs and for at least 6 seconds (Figure 2).

4. Once the test was completed, the participant received an audio and visual illustrating your results.

**Figure 2:**


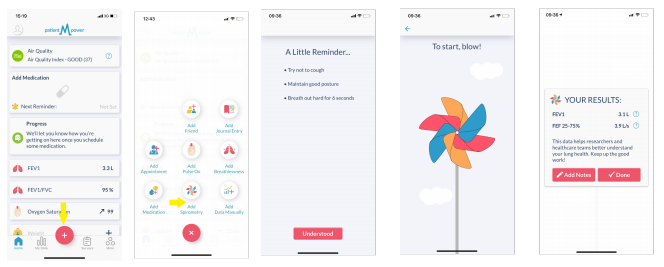


**Using the pulse oximeter**

1. Place the pulse oximeter on your middle finger palm facing up.

Remember before and during using the pulse oximeter;

- Rest for 5 minutes before taking a measurement.
- Ensure your fingers are warm.
- Ensure your fingers are clean and dry.
- Remove nail varnish and false nails to ensure an accurate reading.

2. Open the application and press the add measurement button and select “Add Pulse Ox”. Following this a video with audio and visual content would play describing how to complete the measurement using the device.

3. Hold still until the measurement is completed

4. Once the test was completed, the participant received an visual of their measurement.

5. Following the measurement you are asked “Are you on oxygen?” you should answer this question (Figure 3).

**Figure 3:**


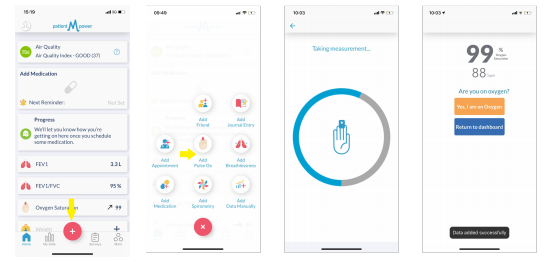


**Inputting data on the self-management programme app**

- Open the application and press the add measurement button and select “Add data manually”.
- You can record measurements taken at home such as temperature you can also add

measurements you recorded at your clinic appointments.

**Breathlessness Score**

Please note rest for 5 minutes before taking a measurement.

1. Press the add measurement button and select “Add breathlessness”.

2. Answer the following questions (Figure 4).

**Figure 4:**
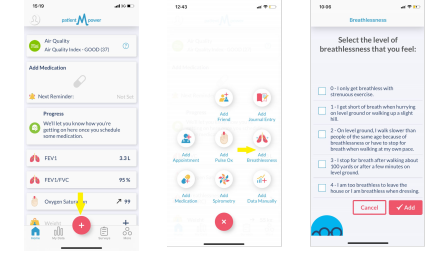


For technical support regarding the app please contact [support@patientmpower.com](mailto:support@patientmpower.com) or call 01 903 8558.
