# Supplementary material for: A Smartphone App Self-Management Program for Chronic Obstructive Pulmonary Disease: Randomized Controlled Trial of Clinical Outcomes
Source: JMIR Mhealth Uhealth. 2025 Apr 23;13:e56318. doi: 10.2196/56318 (PMC12059498; doi:10.2196/56318)
Supplement: Multimedia Appendix 3 [file mhealth_v13i1e56318_app3.docx]

# Sensitivity analysis

|  | **Control=No exacerbation, Exacerbation otherwise** | | | | **Control=Exacerbation, No exacerbation otherwise** | | | |
| --- | --- | --- | --- | --- | --- | --- | --- | --- |
|  | Arm 1 | Arm 2 | Arm 3 | **P-value** | Arm 1 | Arm 2 | Arm 3 | **P-value** |
| **Hospital**  **Six months**  Yes  No | 0.39  0.32 | 0.11  0.39 | 0.50  0.28 | 0.059 | 0.20  0.36 | 0.13  0.38 | 0.67  0.26 | 0.011 |
| **Twelve months**  Yes  No | 0.42  0.31 | 0.29  0.35 | 0.29  0.34 | 0.629 | 0.26  0.36 | 0.32  0.34 | 0.42  0.30 | 0.582 |
| **GP** |  |  |  |  |  |  |  |  |
| **Six months**  Yes  No | 0.41  0.28 | 0.21  0.43 | 0.38  0.28 | 0.071 | 0.33  0.34 | 0.22  0.41 | 0.44  0.25 | 0.088 |
| **Twelve months**  Yes  No | 0.33  0.34 | 0.27  0.41 | 0.40  0.25 | 0.246 | 0.26  0.41 | 0.28  0.39 | 0.47  0.20 | 0.028 |
|  |  |  |  |  |  |  |  |  |
