# Supplementary material for: A Smartphone App Self-Management Program for Chronic Obstructive Pulmonary Disease: Randomized Controlled Trial of Clinical Outcomes
Source: JMIR Mhealth Uhealth. 2025 Apr 23;13:e56318. doi: 10.2196/56318 (PMC12059498; doi:10.2196/56318)
Supplement: Multimedia Appendix 4 [file mhealth_v13i1e56318_app4.docx]

Summary statistics for secondary outcomes

| **Variable** | **Baseline** | **Six months** | **Twelve months** |
| --- | --- | --- | --- |
| **Exercise capacity**  Excellent  Good  Limited  Poor | 6 (6.52)  35 (38.04)  22 (23.91)  29 (31.52) | 7 (8.05)  39 (44.83)  30 (34.48)  11 (12.64)  Missing = 5 | 7 (8.24)  37 (43.53)  28 (32.94)  13 (15.29)  Missing = 8 |
| **HRQoL**  Very good control  moderate control  Poor control  Extremely poor control | 3 (3.26)  25 (27.17)  53 (57.61)  11 (11.96) | 12 (13.79)  43 (49.43)  29 (33.33)  3 (3.45)  Missing =5 | 8 (9.41)  51 (60.00)  20 (23.53)  6 (7.06)  Missing =8 |
| **Self-efficacy**  Not at all confident  very little confidence  little confidence  confident  very confident | 10 (10.87)  18 (19.57)  30 (32.61)  22 (23.91)  12 (13.04) | 5 (5.75)  6 (6.90)  32 (36.78)  18 (20.69)  26 (29.89)  Missing = 5 | 3 (3.3)  13 (15.4)  22 (26.2)  22 (26.2)  24 (28.6)  Missing =8 |
| **Step count**  <100  100-300  300-500  500-1000  1000-3000  3000-5000  5000-10000  >10000  Unknown  Others | 1 (1.1)  0(0)  1 (1.19)  3 (3.3)  8 (8.7)  18 (19.6)  18 (19.6)  7 (7.6)  33 (35.9)  3 (3.3) | 1 (1.14)  0 (0.00)  0 (0.00)  2 (2.27)  10 (11.36)  24 (27.27)  11 (12.50)  11 (12.50)  27 (30.68)  2 (2.27)  Missing = 5 | 2 (2.33)  1 (1.16)  2 (2.33)  5 (5.81)  10 (11.63)  15 (17.44)  23 (26.74)  10 (11.63)  17 (19.77)  1 (1.16)  Missing = 8 |
